# Epidemiology of Viral Hepatitis from 2007 to 2016 in Karbala Governorate, Iraq

**Published:** 2019-06-11

**Authors:** Mohammed A. Merzah, Ali Abd Al-Latif G Mohammed, Ali Neamah Hasan Al-Aaragi, Maytham Salim

**Affiliations:** ^1^Lecturer at Department of Community Health, Technical Institute of Karbala, Al-Furat Al-Awsat Technical University- Kufa, Iraq; ^2^Master of Community Health, Assistant Lecturer at the Department of Community Health, Technical Institute of Karbala, Al-Furat Al-Awsat Technical University- Kufa, Iraq

**Keywords:** Hepatitis, Iraq, Prevalence, Migrant

## Abstract

**Background:** An escalation, as three times more, had been recognized in cases of hepatitis A (HAV) from 2009 to 2014 among Iraqi people. Regarding hepatitis B and C, Iraq is considered as a low endemic country comparing to neighbors.

**Study design:** A retrospective cohort study.

**Methods:** Data incorporated from 2007 to 2016 were collected through a federal survey conducted by the Health Directorate of Karbala, who administrates all hospitals (three public hospitals and five private hospitals) and 40 health centers in Karbala City, Iraq. The four types of hepatitis and demographic information of all cases were included.

**Results:** A vivid shifting in the prevalence of HAV showed a decreasing pattern, that is, from 632 cases (PR=61) in 2007 to 314 cases (PR=33) in 2008. In 2012, its prevalence was twice greater (695 cases, PR=63.2). The PR of HBV also changed from 52 cases (PR=5.8) in 2007 to 26 cases (PR=2.8) in 2008. Regarding HCV, a decreasing pattern with 13 cases (PR=1.4) in 2007 and 12 cases (PR=1.2) in 2009 was seen. This number increased to 60 cases (PR=3.9) in 2016. For HEV, more cases were reported (47 cases, PR=4.7) in 2010.

**Conclusion:** The four types of hepatitis have been highly prevalent since 2010. The high number of migrants to Karbala Governorate and unavailability of immunization might be reasons behind the high prevalence of the four-types of hepatitis.

## Introduction

Viral hepatitis has remained a major public health problem worldwide. With advancements in technologies, eight distinct types of hepatitis viruses have been described: hepatitis A, B, C, D, E, G, TT and SEN viruses ^1^. All these types may cause death or outbreak ^2^. Hepatitis type B (HBV) and hepatitis type C (HCV) are categorized under blood transmission diseases. However, hepatitis type A (HAV) and hepatitis type E (HEV) are classified under poor hygiene diseases. Hepatitis type D (HDV) is considered a subviral satellite because its existence relies on the presence of HBV ^3^. Hepatitis cases have increased during the last decade among Iraqi people. An escalation, as three times more, had been recognized in cases of HAV from 2009 to 2014. Regarding HBV and HCV, Iraq is considered as a low endemic country comparing to neighbors. Increasing the incidence of all types of hepatitis in Iraq may be due to security situation and overcrowding of refugees and migrants; and thus, lack of availability of vaccination ^4^.

HBV and HCV have a higher prevalence than other types of hepatitis in developed countries. People who suffer from either HBV or HCV may develop hepatitis cirrhosis because of chronicity that results from infection by type B or type C. Approximately, 7% of the total world population is a chronic carrier of HBV, although HCV infection is found in 160 million individuals, which represent 3% of the total world population ^3^. The prevalence rates (PRs) of HBV and HCV in Iraq were 1.6% and 0.4% in 2006, respectively ^2^.

HAV and HEV are the most common types of liver infection in developing countries ^3^. They are commonly transmitted through contaminated water and food. HAV and HEV occasionally occur as sporadic cases in industrialized countries. The concurrency of HAV and HEV is observed in people who have pets at home or consume liver meat frequently. In developing countries, poor health education, sanitation and personal hygiene are the common factors that increase the prevalence of HAV and HEV ^3^.

Only HBV and HCV have been studied in Iraq, which is a middle-income country, due to their transmission. Other hepatitis types have been poorly described by Iraqi scholars, although they are highly prevalent amongst the Iraqi population. The current political situation in Iraq may contribute to the high incidence of such diseases. For instance, Karbala City, 100 km south of Baghdad, is one of the cities that attract refugees after the fall of the regime. The increasing population density in Karbala City is the main cause of the spread of different diseases.

The routine surveillance system of hepatitis in Iraq was a priority of the Ministry of Health, to build the capacity of health personnel to cope with patients. Providing medicines, diagnostic services, and availability of immunization were sole parts of the surveillance system ^3^.

We aimed to calculate the PR of hepatitis types and their relationship with other demographic data, and to detect the factors that related to the accelerating in prevalence rate (PR) of hepatitis.

## Methods

After obtaining the official agreement from human right directorate, data incorporated from 2007 to 2016 were collected through a federal survey conducted by the Health Directorate of Karbala, who administrates all hospitals (three public hospitals and five private hospitals) and 40 health centers in Karbala City. Hepatitis A, B, C, and E and demographic information of all cases were included in this study. A federal survey, which was a retrospective observational study using the secondary data, was a part of a national study designed to provide national statistics on the health of Iraqi civilians. The national survey was conducted in all 18 Iraqi governorates; both urban and rural areas were included. Karbala has been chosen by the researchers due to the high migrants who were come to this city comparing to another governorate.

We estimated period prevalence by using number of cases for each type of hepatitis as nominator and the number of population at a specific year as denominator ^5^. Number of population of each year starting from 2007 to 2016 was acquired from Ministry of Planning and Office of Migrants at Karbala City ^6^.

## Results

Increasing number of migrants’ families in Karbala City has an impact on the health stats as some of health authorities emphasized. Laboratories under the Health Directorate of Karbala reported 7,044 cases of viral hepatitis from 2007 to 2016. In terms of gender, the distribution of hepatitis virus in males was more than females. In particular, the distributions of HAV and HBV were 58% and 71%, respectively. Females were highly affected by HCV and HEV.

Amongst the hepatitis types, HAV was the most frequently reported (n=6060, 86%). Most of the infected people were in their early childhood aged 10 yr or less. About 58% of HAV cases were among migrants people.

The PR (as calculated per 100,000 populations) of the four types of hepatitis in Karbala City have largely changed over the last 10 years. The shift in the prevalence of HAV showed a decreasing pattern, that is, from 632 cases (PR=61) in 2007 to 314 cases (PR=33) in 2008. In 2012, its prevalence was twice greater (695 cases, PR=63.2). The PR of HBV also changed from 52 cases (PR=5.8) in 2007 to 26 cases (PR=2.8) in 2008. Its PR then increased and reached 101 cases (PR=6.7) in 2016. For HCV, we noticed a decreasing pattern with 13 cases (PR=1.4) in 2007 and 12 cases (PR=1.2) in 2009. This number increased to 60 cases (PR=3.9) in 2016. For HEV, more cases were reported (47 cases, PR=4.7) in 2010 (Table 1).

**Table 1:** Prevalence rate of viral hepatitis reported per year in Karbala, Iraq, from 2007 to 2016

**Year**	**No. of** **Population**	**No. of Migrants’** **Families**	**Prevalence rate per 100,000 population**							
			**Hepatitis A**		**Hepatitis B**		**Hepatitis C**		**Hepatitis E**	
			**Number**	**Percent**	**Number**	**Percent**	**Number**	**Percent**	**Number**	**Percent**
2007	891,305	1350	632	70.9	52	5.8	13	1.4	0	0.0
2008	948925	1429	314	33.0	26	2.8	13	1.4	0	0.0
2009	1000000	1512	250	25.0	21	2.1	12	1.2	4	0.4
2010	1014218	1652	549	54.0	29	3.0	17	1.6	47	47
2011	1048128	1678	684	65.0	65	6.2	27	2.5	8	1.1
2012	1100000	1711	695	63.2	72	6.6	29	2.6	7	0.6
2013	1099217	12320	722	65.7	81	7.4	32	2.9	7	0.6
2014	1098718	13800	730	66.4	87	7.9	37	3.4	3	0.3
2015	1461367	12726	719	49.2	93	6.4	40	2.7	1	0.1
2016	1508143	11820	765	50.8	101	6.7	60	3.9	0	0.0

A huge shift in number of migrants’ families during the years 2012 to 2016 has a companying with elevating in prevalence rate of HAV, HBV, and HCV. Coefficient correlation was found significant between number of migrant’s families and each of HBV and HCV cases with values 0.811, *P*-value=0.004 and 0.785, *P*-value= 0.007, respectively. This means increasing in number of migrants’ families had an effect on increasing in HBV and HCV cases from 2007 to 2016.

**Figure 1:**
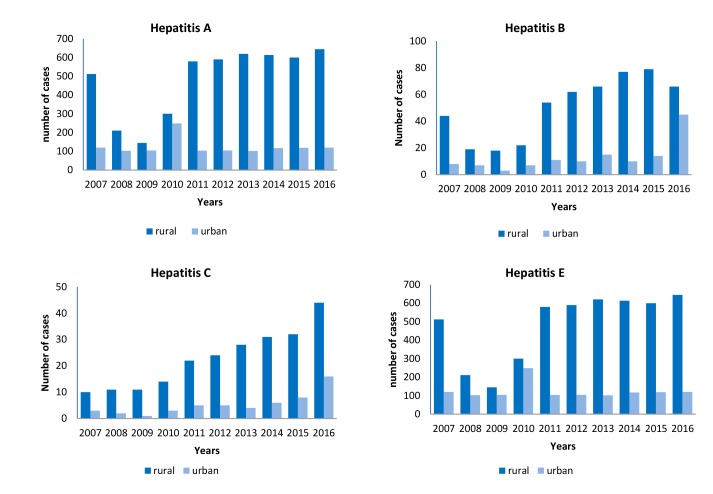
Total number of viral hepatitis cases by residency

The number of rural regions affected by the four types of viral hepatitis in Karbala Governorate was higher than in urban regions. Figure 1 shows that the majority of HAV cases were reported from urban areas (n=50 cases) in 2010. In 2016, the majority of HBV cases were reported from both urban and rural areas, with 66 cases and 45 cases, respectively. For HCV, all of the cases were almost equally reported from both regions throughout the 10 years that covered this study. HEV cases were mostly reported from urban areas in 2010 (45 cases).

**Figure 2:**
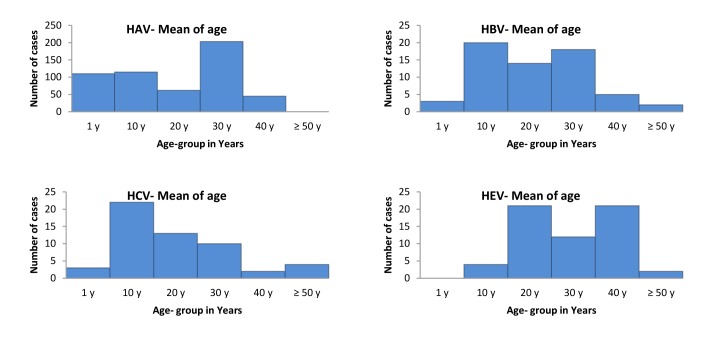
Total number of viral hepatitis cases by age group

HAV cases were mostly detected in the age group of 30–40 yr and the total number of cases was 203, followed by 115 cases in the age group of 10–20 yr and 110 cases in the age group of ≥10 years. HBV (20 cases) and HCV (22 cases) highly affected the age group of 10–20 years. HEV affected the age groups of 20–30 and 40–50 yr with 21 cases in each group (Figure 2).

## Discussion

“Hepatitis A is an acute, typically self-limiting liver disease and one of the most common infectious diseases in the world” ^7^. HAV was highly prevalent during the 10 years when this research was performed. Poor personal hygiene and health education might be the main reason for the increasing number of HAV cases. Most of migrants’ families lived in camps far away from the city center where poor sanitations and limited access to safe drinking water. This may contribute to the incidence of HAV ^3, 7^.

The prevalence of HCV increased from 1.4 to 3.9 during the last decade. However, the HCV incidence is slightly higher in other neighboring countries comparing to Karbala Province, Iraq ^4, 8^. For HBV, the prevalence decreased in 2008–2010 because of the effectiveness of a vaccination program, the one that mandated all six-years-old to be vaccinated against HBV as a requirement for entering school. The prevalence of HBV is also lower than previously reported due to the effectiveness of the ongoing nationwide vaccination program, especially for HBV that shows a steadily decreasing incidence correlated with a remarkably positive effect on its annual incidence; the introduction of new and more effective healthcare facilities also plays an important role in this achievement ^9-11^. Since 2011, the prevalence of HBV has increased continuously because the high migration to Karbala due to ISIL attack in some north-cities of Iraq, leads to the inability of the health sector to cover all people with vaccination.

The PR of HBV during the last 10 years was higher than that in other states in the same region. The PRs of HBV amongst Kuwaiti nationals and non-Kuwaiti Arabs were 1.1% and 3.5%, respectively^12^. Males were higher in having HBV than females. This finding is corresponding with studies conducted in Pakistan and Bangladesh, which showed the prevalence of HBV in males, was higher than females ^4, 13^. In comparison with males in other age groups, those aged above 40 yr are more likely to have HBV ^14^. Males were more likely to be infected with HAV and HBV than females, whereas HEV and HCV were more common amongst females than males. This finding was inconsistent with research conducted in Pakistan, where HAV (69.5%) and HEV (72.4%) were more common in males than in females ^14^, possibly because of a change in age distribution, especially for HAV.

Few community-based studies have been conducted to estimate the incidence and prevalence of HAV and HEV in Iraq. Our study confirmed that HAV most commonly affected the children, and more than 60% of HAV cases were patients younger than 14 years. By comparison, HBV most commonly affected the adults, and 69% of HBV cases were patients aged 15–44 years. HCV is most commonly detected in the elderly, and more than 59% of HCV cases are patients older than 45 yr^15^. HCV is leading to hepatic inflammation in addition to untiring systemic inflammation, and the risk would be greater with aging. The risk of liver disease in the setting of HCV has increased with aging-related mechanisms. Elderly are highly vulnerable to disease, especially HCV, due to impaired immunity, decline in the ratio of liver flow^16^.

The prevalence of the four types of hepatitis amongst residents of rural areas is higher than that of urban areas. Poor sanitation system, limited access to healthy drinking water, and low health-education amongst residents of rural areas result in increasing number of cases of HAV and HEV ^17^. HBV and HCV were highly prevalent in rural areas ^15, 18-22^.

Number of migrants to Karbala was increased continuously during the last decade. About 1350 families were migrated to Karbala in 2007. This number was enormously increased in 2014 to reach 13800 families ^6^. More than 10000 migrant families did not have access to health services due to losing their IDs and official documents. More than 2000 migrant families shared one shelter/ camp for each 3-5 families. More than 5000 kids were unable to join schools due to living in remote areas ^6^. All these might be reasons behind the unceasingly raising in number of health issue, especially hepatitis cases. As a retrospective study, a limitation of assessing temporal relationship is difficult to measure, but we avoided this limitation through including large sample.

## Conclusion

The four types of hepatitis (Hepatitis A, B, C, and E) have been highly prevalent since 2010. HAV and HBV have been decreased during the years 2008 and 2009 and then elevated again in 2010. The high number of migrant families to Karbala Governorate might be a reason four types of hepatitis during the last decade. Bad security situation and accelerating number of refugees and migrants were led to an availability of immunization; and thus, levitation the incidence rate of hepatitis.

## Acknowledgements

We appreciate the sincere help provided by Health Directorate of Karbala City, especially the Department of Diseases Control by providing the data of this research.

## Conflict of interest

The authors declare that there is no conflict of interests.

## Funding

There is no resource of funding.

## Highlights

- Migrants might be a reason to accelerate hepatitis cases.
- HCV was high prevalent among elderly.
- HBV and HCV were high prevalent in rural areas.
